# The effect of a previous created distal arteriovenous-fistula on radial bone DXA measurements in prevalent renal transplant recipients

**DOI:** 10.1371/journal.pone.0200708

**Published:** 2018-07-26

**Authors:** Anna Walder, Martin Müller, Suzan Dahdal, Daniel Sidler, Vasilios Devetzis, Alexander B. Leichtle, Martin G. Fiedler, Albrecht W. Popp, Kurt Lippuner, Bruno Vogt, Dominik Uehlinger, Uyen Huynh-Do, Spyridon Arampatzis

**Affiliations:** 1 Department of Nephrology and Hypertension, Inselspital, Bern University Hospital and University of Bern, Bern, Switzerland; 2 Department of Emergency Medicine, Inselspital, Bern University Hospital and University of Bern, Bern, Switzerland; 3 Center of Laboratory Medicine, Inselspital, Bern University Hospital and University of Bern, Bern, Switzerland; 4 Department of Osteoporosis, Inselspital, Bern University Hospital and University of Bern, Bern, Switzerland; Istituto Di Ricerche Farmacologiche Mario Negri, ITALY

## Abstract

**Background:**

Accelerated bone loss occurs rapidly following renal transplantation due to intensive immunosuppression and persistent hyperparathyroidism. In renal transplant recipients (RTRs) due to the hyperparathyroidism the non-dominant forearm is often utilized as a peripheral measurement site for dual-energy x-ray absorptiometry (DXA) measurements. The forearm is also the site of previous created distal arteriovenous fistulas (AVF). Although AVF remain patent long after successful transplantation, there are no data available concerning their impact on radial bone DXA measurements.

**Methods:**

In this cross-sectional study we performed DXA in 40 RTRs with preexisting distal AVF (RTRs-AVF) to assess areal bone mineral density (aBMD) differences between both forearms (three areas) and compared our findings to patients with chronic kidney disease (CKD, n = 40), pre-emptive RTRs (RTRs-pre, n = 15) and healthy volunteers (n = 20). In addition, we assessed relevant demographic, biochemical and clinical aspects.

**Results:**

We found a marked radial asymmetry between the forearms in RTRs with preexisting AVF. The radial aBMD at the distal AVF forearm was lower compared to the contralateral forearm, resulting in significant differences for all three areas analyzed: the Rad-1/3: median (interquartile range) in g/cm^2^, Rad-1/3: 0.760 (0.641–0.804) vs. 0.742 (0.642, 0.794), p = 0.016; ultradistal radius, Rad-UD: 0.433 (0.392–0.507) vs. 0.420 (0.356, 0.475), p = 0.004; and total radius, Rad-total: 0.603 (0.518, 0.655) vs. 0.599 (0.504, 0.642), p = 0.001). No such asymmetries were observed in any other groups. Lower aBMD in AVF forearm subregions resulted in misclassification of osteoporosis.

**Conclusions:**

In renal transplant recipients, a previously created distal fistula may exert a negative impact on the radial bone leading to significant site-to-site aBMD differences, which can result in diagnostic misclassifications.

## Introduction

Accelerated bone loss occurs rapidly following renal transplantation and is associated with considerable fracture risk compared to other chronic kidney disease (CKD) patients [1,2]. During the post-transplant period the major skeletal effects of intense immunosuppression and persistent hyperparathyroidism are cortical and trabecular bone loss [3–5]. Within the first five years after transplantation, more than one quarter of renal transplant recipients (RTRs) will sustain a fracture, most commonly a low-energy fracture of the extremities [6,7]. Consequently, evaluation by dual-energy X-ray absorptiometry (DXA) and classification of bone loss at peripheral bone sites are critical for fracture risk assessment and recommended by current guidelines [8–10].

In clinical practice areal bone mineral density (aBMD) measured by DXA, is the gold standard for diagnosing bone disease. There is also growing evidence that aBMD by DXA predicts fractures across the spectrum of CKD severity [10,11]. Distal radius, a peripheral cortical-rich bone, is one of the preferred measuring site for assessing catabolic skeletal effects of hyperparathyroidism and is also predictive for fracture risk in CKD patients [12–16]. The non-dominant forearm is often utilized as a peripheral measuring site in renal transplant recipients with persistent hyperparathyroidism. The distal forearm is also the preferential site for radiocephalic arteriovenous-fistulas (AVF), which serves as the standard vascular access site for hemodialysis. AVF remain patent in most RTRs long after discontinuation of hemodialysis following successful transplantation. Data on the potential long-term impact of AVF on peripheral bones are scarce and currently available only for hemodialysis patients [17]. No studies in RTRs have been ever conducted investigating the influence of a previously created radiocephalic AVF on peripheral radial bone aBMD measurements and potential diagnostic misclassifications. Thus, the main objective of this cross-sectional study was to explore the impact of the radiocephalic AVF on the ipsilateral radial bone characteristics in RTRs. We conducted aBMD measurements between both forearms in prevalent RTRs with previously created radiocephalic AVF and compared them with forearms measurements in relevant groups including pre-emptive renal transplant patients (RTRs-Pre), CKD-patients with no history of dialysis or AVF placement as well as healthy volunteers.

## Subjects and methods

### Study participants

All study participants, were recruited within the Department of Nephrology and Hypertension, Bern Inselspital University Hospital, Switzerland. All eligible subjects were 18 years of age and CKD was defined as an estimated glomerular filtration rate (eGFR) of ≤ 90 mL/min. RTRs included in our study had documented, stable renal function during one month before study recruitment. Healthy controls (normal kidney function according to age related eGFR calculation, with no microalbuminuria or antihypertensive medications) were recruited among the staff of the Nephrology department. The eGFR was determined by CKD-EPI formula [18].

RTRs with AVF were on hemodialysis for at least six months before transplantation. Radial AVF in RTRs were located on the same arm during the dialysis period. Preemptive RTRs were transplanted before initiation of maintenance dialysis procedure or AVF surgical creation. Patients with factors that might cause a difference in aBMD between the two forearms that could interfere with results were excluded from the study (i.e. previous forearm fracture, bilateral AVF, vascular stent, or extra osseous calcification based on radiological/DXA examinations). The Ethics Committee of the Canton of Bern, Switzerland, approved the primary study protocol and the subsequent amendments (KEK- 082/12, amend. 02/2017). Written informed consent was obtained from all participants.

### Clinical assessments

All patients included in the study were surveyed regarding medical history, medication use, history of forearm fractures and previous glucocorticoid exposure. A fragility fracture was defined as a fracture associated with trauma equivalent to or less than a fall from standing height. Medical records were reviewed to assess the primary kidney disease, current medication including 25-hydroxyvitamin D/calcium supplementation as well as reports of AVF surgery, hemodialysis duration, and transplantation data, where applicable. When available, reported fractures were confirmed with X-ray reports. In all subjects, height was measured twice on a wall-mounted Harpenter Stadiometer^TM^ and weight on a calibrated scale.

### Laboratory assessment

Each subject reported to the laboratory for blood sampling after fasting overnight. Serum creatinine, calcium and phosphorus analyses were performed using automated techniques (Roche Modular P800, Roche Diagnostics, Manheim, Germany). Serum iPTH was determined using an electrochemiluminescence immunoassay (Cobas, Roche Diagnostics, Mannheim, Germany). Serum 25-hydroxyvitamin D was measured by radioimmunoassay (Gamma B, Immunodiagnostics Systems LTD, Boldon, UK).

### Assessment of areal bone mineral density

Areal BMD was measured at the Department of Osteoporosis, University Hospital Bern, Switzerland, by DXA. Scans were performed using a standard DXA device (Hologic QDR 4500 A^TM^, Hologic, Bedford, MA, USA). BMD was measured at three sites including the distal radius (first third: Rad-1/3, ultradistal: Rad-UD, total: Rad-total) subregions. The manufacturer's software was used to analyze the regions of interest at the proximal femur and the distal radius. Values of aBMD were expressed in g/cm^2^. Validation of assay precision was performed daily using an artificial anthropometric spine supplied by the manufacturer. The overall precision error was 0.3% in vitro, and the mean precision error in vivo in our hands was 1.1%- 1.4%.

### Statistical analysis

Statistical analyses were performed using Stata® 13.1 (StataCorp, College Station, Texas, USA). Continuous variables of clinical characteristics and covariates of all study participants were compared using the Kruskal-Wallis test and Wilcoxon rank-sum test as post-hoc test since most variables were not normally distributed data. Categorical variables were compared using Fisher’s exact test or in case of paired categorical data McNemar’s test. The site-to-site differences in bone density parameters of the forearms (aBMD) were compared within each group using the Wilcoxon signed-rank test. Comparison of aBMD% difference (ΔBMD%) between both forearms among RTRs-AVF (ΔBMD% between AVF vs. non-AVF site) and other groups (ΔBMD% between non-dominant vs. dominant site) were calculated using the following formulas: in RTR-AVF [ΔBMD% = (nonAVF − AVF) / non-AVF × 100)] and for all other groups [ΔBMD% = (dominant–non-dominant) / non-dominant × 100)]. Spearman’s correlation coefficients were calculated to investigate for any association between ΔBMD and AVF. Continuous data are reported as median (interquartile range, IQR) and categorical data are shown as number (percentage) with a level of significance of p < 0.05.

## Results

### Characteristics of the study population

The study population consisted of 40 RTRs with AVF, 40 CKD patients, 15 preemptive RTRs and 20 healthy volunteers. Baseline characteristics concerning demographics, clinical parameters and medications of the study population are shown in **Table 1**.

**Table 1:** Baseline clinical and biochemical characteristics. Values are presented as median (interquartile range) for continuous variables or as absolute number and percentages (%) for categorical variables.

		RTRs-AVF (n = 40)	CKD (n = 40)	Healthy (n = 20)	RTRs-Pre (n = 15)
**Demographics**					
	Age, years	58 (49, 63)	64 (48, 71)	52 (46, 57)	55 (47, 60)
	Male sex	31 (78)	26 (65)	5 (25)	14 (93.3)
	Height, cm	168 (161, 174)	168 (159, 172)	168 (160, 173)	177 (169, 178)
	Weight, kg	75 (63, 88)	76 (54, 89)	69 (60, 86)	82 (74, 94)
	BMI, kg/m^2^	27 (23, 30)	27 (25, 31)	25 (22, 33)	26 (24, 30)
	Patent AV Fistula	26 (65)	-	-	-
	AV Fistula left forearm	31 (78)	-	-	-
	Age at RTx, years	49 (39, 46)	-	-	54 (42, 59)
	Time on dialysis, months	27 (14, 52)	-	-	0
	Time RTx to DXA, months	70 (19, 126)	-	-	27 (8, 56)
**Clinical parameters**					
*Etiology of renal failure*					
	Glomerulonephritis/ vasculitis	14 (35)	16 (40)		7 (47)
	Diabetic nephropathy	6 (15)	3 (8)		1 (7)
	Vascular nephropathy	2 (5)	9 (23)		0 (0)
	Hereditary/APDKD	11 (27.5)	3 (7.5)		1 (6.67)
	Other or unknown	14 (35)	16 (40)		7 (46.67)
*Medication*, *intake*					
	Glucocorticoids	27 (68)	9 (23)	-	13 (87)
	Calcineurin inhibitors	34 (85)	3 (8)	-	15 (100)
	Antimetabolites	33 (83)	2 (5)	-	14 (93)
	Using calcium	16 (40)	12 (30)	3 (15)	5 (33)
	Under vitamin D	25 (63)	18 (45)	2 (10)	9 (60)
**Biochemical parameters**					
	Creatinine	133 (106, 222)	120 (86, 212)	67 (62, 74)	156 (128, 168)*
	eGFR	46 (26, 54)	48 (26, 71)	90 (86, 90)	67 (38, 55)*
	Calcium	2.4 (2.3, 2.5)	2.3 (2.2, 2.4)	2.3 (2.2, 2.4)	2.4 (2.4, 2.5)*
	iPTH	94 (54, 143)	48 (33, 98)	30 (26, 37)	85 (38, 128)*
	25(OH)VitD	42 (28, 54)	52 (32, 64)	58 (46, 78)	59 (43, 65)*
	1,25 (OH)_2_-Vit D	46 (34, 74)	45 (30, 63)	88 (70, 103)	97 (60, 113)*

RTRs: renal transplant recipients, AVF: arterio-venous fistulas, CKD: chronic kidney disease, Healthy: healthy volunteers, RTRs-Pre: preemptive renal transplant recipient, eGFR: estimated glomerular filtration rate, CKD-EPI: chronic kidney disease—epidemiology collaboration, iPTH: intact parathormone, Vit-D: vitamin D. RTRs-Pre: preemptive renal transplant recipient, BMI: body mass index, RTx: renal transplantation, Hx: history, DXA: dual energy x-ray absorptiometry, ADPKD: autosomal dominant polycystic kidney disease.

*statistically significant difference <0.005, (Kruskal-Wallis test)

Due to the heterogeneous groups, no statistical tests were performed concerning baseline characteristics. All RTRs with AVF had been on dialysis for at median of 27 months before renal transplantation. Most of them had a still functioning AVF at the non-dominant forearm. The most common etiology of primary kidney disease in all patient groups was glomerulonephritis. The standard transplantation immunosuppressant regimen at our center consists of quadruple therapy with baxiliximab, calcineurin inhibitors (primarily cyclosporin A), antimetabolites (primarily mycophenolate mofetil), and steroids which are tapered slowly. Baseline laboratory values including intact serum PTH and vitamin D levels are shown in Table 1.

### Evaluation of aBMD between forearm aBMD measurements

aBMD evaluation of the forearm in RTRs revealed a significant and negative impact of AVF to the ipsilateral to AVF radius site (Table 2).

**Table 2:** Comparison between forearms in renal transplant recipients with AVF (RTRs-AVF, n = 40).

Measurement		Non-AVF forearm	AVF forearm	p-value*
**Radius, BMD, g/cm**^**2**^				
	Rad-1/3	0.760 (0.641, 0.804)	0.742 (0.642, 0.794)	0.016^*^
	Rad-total	0.603 (0.518, 0.655)	0.599 (0.504, 0.642)	0.001^*^
	Rad-UD	0.433 (0.392, 0.507)	0.420 (0.356, 0.475)	0.004^*^
**T-Score, SD**				
	Rad-1/3	-1.0 (-1.8, 0.1)	-1.1 (-2.0, -0.4)	0.014^*^
	Rad-total	-1.4 (-2.1, -0.4)	-1.6 (-2.5, -0.8)	0.004^*^
	Rad-UD	-1.5 (-2.0, -0.6)	-1.6 (-2.5, 1.0)	0.002^*^

BMD: bone mineral density, RTRs: renal transplant recipients, AVF: arteriovenous fistula, Rad: radius, 1/3: one-third, Rad-total: total radius, Rad-UD: ultradistal radius.

* statistically significant difference (Wilcoxon signed-rank test), values are presented as median (interquartile range)

The aBMD at the AVF forearm site was significantly lower than at the forearm without AVF. This observation was evident in all subregions of the AVF radius sites, i.e. at the 1/3 Radius, median (IQR); [Rad-1/3: 0.760 (0.641, 0.804) vs. 0.742 (0.642, 0.794), p = 0.016], ultra-distal Radius [Rad-UD: 0.433 (0.392–0.507) vs. 0.420 (0.356–0.475), p = 0.004], and total radius [Rad-total: 0.603 (0.518–0.655) vs. 0.599 (0.504–0.642), p = 0.001]. Accordingly, T-scores in the AVF forearm were also significantly lower in all subregions of the AVF compared to non-AVF radial sites.

Comparison between aBMD forearms subregions (dominant forearm vs. non-dominant forearm) in CKD patients (n = 40, CKD), in the healthy control group (n = 20, Healthy), and in preemptive renal transplantation group (n = 15, RTR-Pre) are shown in Table 3.

**Table 3:** Comparison between aBMD forearms subregions (dominant forearm vs. non-dominant forearm) in chronic kidney disease patients (CKD, n = 40), healthy control group (Healthy, n = 20), and preemptive renal transplantation group (RTRs-pre, n = 15).

		Dominant forearm	Non-dominant forearm	p-value^a^
**CKD (n = 40)**				
BMD, g/cm^2^				
	Rad-1/3	0.753 (±0.132)	0.756 (0.704, 0.823)	0.990
	Rad-total	0.620 (±0.117)	0.623 (0.571, 0.695)	0.326
	Rad-UD	0.474 (±0.101)	0.488 (0.427, 0.533)	0.226
T-Score, SD				
	Rad-1/3	-0.1 (-1.6, 0.8)	0.1 (-1.7, 0.7)	0.922
	Rad-total	-0.3 (-1.2, 0.4)	-0.4 (-1.1, 0.2)	0.402
	Rad-UD	-0.5 (-1.2, 0.0)	-0.5 (-1.3, -0.1)	0.342
**Healthy (n = 20)**				
BMD, g/cm^2^				
	Rad-1/3	0.712 (0.683, 0.740)	0.713 (0.677, 0.784)	0.380
	Rad-total	0.595 (0.530, 0.657)	0.593 (0.552, 0.669)	0.218
	Rad-UD	0.443 (0.376, 0.537)	0.457 (0.396, 0.512)	0.550
T-Score, SD				
	Rad-1/3	0.0 (-0.8, 0.4)	-0.1 (-0.5, 0.6)	0.350
	Rad-total	0.0 (-1.0, 0.9)	-0.1 (-0.8, 0.7)	0.270
	Rad-UD	-0.3 (-1.2, 0.9)	-0.2 (-1.0, 0.8)	0.575
**RTRs-pre (n = 15)**				
BMD, g/cm^2^				
	Rad-1/3	0.758 (0.732, 0.809)	0.769 (0.752, 0.800)	0.712
	Rad-total	0.584 (0.555, 0.618)	0.580 (0.554, 0.629)	0.320
	Rad-UD	0.434 (0.377, 0.444)	0.417 (0.369, 0.462)	0.994
T-Score, SD				
	Rad-1/3	-0.3 (-1.6, -0.1)	-0.8 (-1.2, 0.5)	0.233
	Rad-total	-1.9 (-2.5, -0.7)	-2.1 (-2.6, 1.0)	0.267
	Rad-UD	-1.9 (-2.8, -1.3)	-2.2 (-3.0, -1.3)	0.147

BMD: bone mineral density, AVF: arteriovenous fistula, Rad: radius, 1/3: one-third, Rad-total: total radius, Rad-UD: ultradistal radius.

^a^ statistically significant difference (Wilcoxon signed-rank test), values are presented as median (interquartile range)

All other analyzed groups, including the CKD-, and the healthy control group which had no history of any hemodialysis treatment or the RTR-Pre with no history of AVF surgical creation on either forearm showed no significant differences between the dominant and non-dominant forearm aBMD measurements. Also, no site-to-site differences were observed in T-scores between forearms.

### Evaluation of percent difference of aBMD (ΔBMD%) between forearms and correlation between AVF-use period and ΔBMD%

The comparison of aBMD percentage difference (ΔBMD%) between forearms of all groups (Table 4) were calculated based on the concept that the AVF forearm in RTRs-AVF group corresponds to the non-dominant forearm in subjects with CKD, healthy patients, and preemptive transplanted patients. In all studied subregions (Rad-1/3, Rad-total, and Rad-UD), there was at least a tendency (p<0.1) of a different distribution of the medians of ΔBMD% between the four studied groups. Apart from preemptive transplanted patients, the median of RTR patients was significantly higher compared to the median of ΔBMD% in the healthy group and CKD group in all subregions.

**Table 4:** Comparison of BMD percentage difference (ΔBMD%) between forearms in (RTRs-AVF, n = 40) and i) chronic kidney disease patients (CKD, n = 40), ii) healthy control group (Healthy, n = 20), as well as iii) preemptive performed renal transplantation group (RTR-Pre, n = 15).

Radius measurement ΔBMD%, median (interquartile range)						
	Rad-1/3	p-value^a^	Rad-total	p-value^a^	Rad-UD	p-value^a^
RTRs-AVF	1.2 (-1.3, 4.8)	-	1.8 (-1.1, 9.3)	-	4.4 (-0.8, 9.1)	-
CKD	0.5 (-3, 3.1)	0.070	-1.1 (-2.6, 1.9)	0.003	0.8 (-2.4, 4.6)	0.048
Healthy	-1.7 (-3.5, 2.4)	0.016	-1.4 (-4.2, 1)	0.005	-2.4 (-5.3, 5)	0.021
RTRs-Pre	0 (-3.3, 1.6)	0.108	0.9 (-1.7, 2.5)	0.249	2.4 (-3.8, 7.9)	0.42
p-value^b^	0.0711		0.0066		0.0611	

BMD: bone mineral density, CKD: chronic kidney disease, RTRs: renal transplant recipients, Rad: radius, 1/3: one-third, Rad-total: total radius, Rad-UD: ultradistal radius. ΔBMD% in CKD, Healthy, RTRs-pre: [(dominant–Non-dominant)/Non-dominant] x 100, ΔBMD% in RTRs: [(Non-AVF–AVF)/AVF] x 100

^a^p-value of Wilcoxon rank-sum test to RTRs

^b^p-value of Kruskal-Wallis test between all groups.

Finally, no significant correlation was found by analyzing the impact of the time period between AVF surgical creation until renal transplantation (AVF-use) to the magnitude of the radial site difference (ΔBMD%) in RTRs-AVF; ΔBMD% RAD-1/3: p = 0.960, spearman's rho = -0.0082; ΔBMD% Rad-Total: p = 0.853, spearman's rho = -0.0303; ΔBMD% Rad-UD: p = 0.998, spearman's rho = 0.0006.

**Fig 1:**
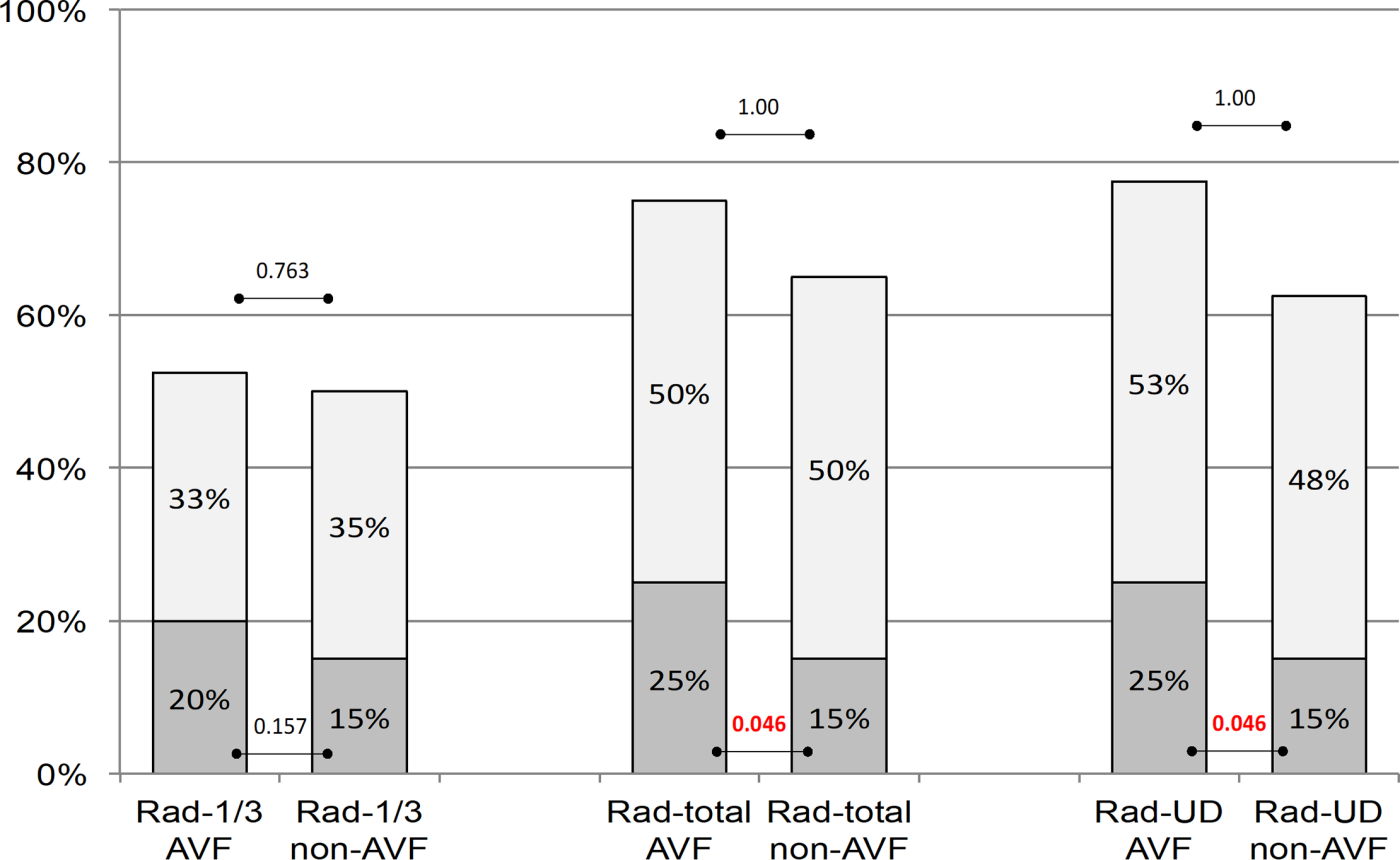
Categorized aBMD findings (%) by T-score ≤-2.5 SD (osteoporosis in dark grey) and T-score >-2.5 & ≤-1 SD (osteopenia in light grey) in renal transplant recipients with AVF as assessed by measurements in all 3 peripheral bone regions (Rad: radius, 1/3: one-third, Rad-total: total radius, Rad-UD: ultradistal radius.) of either forearm (AVF forearm: arteriovenous fistula forearm; non AVF forearm: non-arteriovenous fistula). P-values of McNemars test between the arms.

Discrepancy between classification of osteoporosis and osteopenia by using aBMD assessments at the AVF forearm sites in RTRs with AVF. Fig 1 shows the diagnostic misclassification of aBMD (osteoporosis defined as a T-score ≤ 2.5 SD and osteopenia T-score >-2.5 & ≤-1 SD) in RTRs-AVF as assessed by measurements in different radial subregions of either forearm.

## Discussion

Due to the previous longstanding history of CKD, persistent post transplantation hyperparathyroidism, high doses corticosteroids after transplantation and various mineral and bone disorders virtually all RTRs are vulnerable to rapid cortical and trabecular bone loss. Thus, early evaluation of peripheral cortical bone sites by DXA, particularly in RTRs with longstanding hyperparathyroidism, is crucial for accurate fracture risk assessment and therapy initiation. Our study set out to analyze the impact of a preexisting forearm AVF on the radius of RTRs. A marked radial asymmetry was found in RTRs with preexisting AVF between the site ipsilateral to the AVF compared to the contralateral site, resulting in significant differences between all forearm subregions analyzed including Rad-1/3, Rad-UD, and Rad-total. In contrast, no such asymmetries were observed between radial aBMD measurements of forearms in preemptive RTRs, CKD patients or healthy subjects. Furthermore, lower aBMD at the AVF forearm subregions caused diagnostic misclassification and resulted in more osteoporotic cases.

Although this is the first study reporting the negative impact of the aBMD of the distal radius in RTRs, our findings are in accordance with a previous study conducted in long-term hemodialysis patients. Muxi et al. analyzed 30 long-term end stage renal disease patients on hemodialysis and found that the ipsilateral to AVF forearm had significantly lower aBMD values than the contralateral site in all locations analyzed [17]. A marked peripheral bone asymmetry between the dominant and non-dominant radius has been also shown in active tennis players exposed to asymmetrical mechanical loading, particularly in those who started playing during pre- or early puberty [19,20]. In the general population, as in our healthy control group, numerous studies have reported no site-specific differences for the densitometric properties of the radius [21–24]. Consequently, based on current findings, site-to-site radial differences within the general population seems to be negligible, and whilst measurable in some athletes, is considered exercise-induced. In hemodialysis and transplant patients with a peripheral vascular access the site-specific decrease in radial density is probably attributable to the local effects of the AVF. Moreover, these negative effects on radial bone seem to be AVF specific since radial aBMD asymmetry was not present in preemptive RTRs.

Although the exact pathomechanism behind the site-specific AVF-induced reduction in radial aBMD is unknown, several studies have indicated that blood flow changes can negatively influence the bone geometry of the extremities [25–27]. Possible mechanisms for this include aberration of blood flow to adjacent bones resulting in subsequent hypoperfusion and elevated sympathetic tone. Bone remodeling secondary to the pulsating pressure of an AVF or fistula-pseudoaneurysms may also lead to significant bone loss and erosion of nearby bones, similar to vertebral body lesions resulting from pulsating abdominal aneurysms [28–30]. In our study no significant correlation was found by analyzing the impact of the time period between AVF placement until renal transplantation (AVF-use) to the magnitude of the radial site difference. In addition, several other factors such as prolonged immobilization, underuse of the fistula arm, and the severity of CKD-associated hyperparathyroidism can alter the bone structure of the forearm bone and have a negatively impact on bone mass measurements [31,32]. PTH excess in patients with already established CKD is also characterized by site-specific skeletal effects concerning both bone density and structure [33,34].

The diagnostic usefulness of DXA for fracture risk prediction in the CKD population has long been under debate. Growing evidence from several trials suggests that low aBMD measured by DXA at the radius and hip can predict future fractures in patients with predialysis CKD, on hemodialysis and in RTRs [11,35–37]. However, our findings suggest that assessing aBMD at the radial site ipsilateral to AVF can result in diagnostic misclassification due to a significant and site-specific reduction in aBMD. If such AVF-related differences are already detectable with a ‘low resolution’ method such as DXA, then results derived from high-resolution peripheral quantitative computed tomography (HR-pQCT) could be far more erroneous. As our results agree with previous findings from patients under hemodialysis, measurement of the contralateral side to the AVF, or assessment of both forearms in patients with impaired renal function and secondary hyperparathyroidism should be considered. This is particularly important in research and in clinical settings as well.

Our study does have some limitations. Most importantly, this was a cross-sectional trial and therefore it is not possible to definitively conclude that AVF causes the asymmetry in radial bone density. Several factors other than the AVF which can affect bone characteristics, such as activity levels, lifestyle and dietary factors, were not addressed. Also the dominant forearm may have an impact on radial bone characteristic, an effect described in the literature mostly among highly trained athletes. This study included predominantly a Caucasian cohort of ambulatory dwelling subjects, with a small number of individuals with preemptive transplantation performed, most of them with secondary hyperparathyroidism and immunosuppression according to our local protocols, potentially limiting the translation of our findings to other ethnic groups and RTR cohorts under steroid-sparing regiments. On the other hand, possible local interferences were canceled out by patient selection criteria and contralateral forearm evaluation on the same person. The group selection including a healthy group and RTRs without any history of long term hemodialysis treatment or AVF placement further support our findings although we didn't analysed central skeletal measuring sites.

In conclusion, our findings suggest that in RTRs a previously placed AVF may exert a negative impact on the ipsilateral radius resulting in site-to-site aBMD differences, which are detectable by DXA and can result in diagnostic misclassification. Measurement of both forearms should be considered in RTRs with preexisting AVF in order to avoid diagnostic misclassifications. Longitudinal studies are required to determine the mechanism of AVF-induced radial bone deterioration.

## References

[1] Stehman-Breen CO, Sherrard DJ, Alem AM, Gillen DL, Heckbert SR, Wong CS, et al (2000) Risk factors for hip fracture among patients with end-stage renal disease. Kidney Int 58: 2200–2205. 10.1111/j.1523-1755.2000.00394.x 11044242

[2] Ball AM, Gillen DL, Sherrard D, et al (2002) RIsk of hip fracture among dialysis and renal transplant recipients. JAMA 288: 3014–3018. 12479766 10.1001/jama.288.23.3014

[3] Rajapakse CS, Leonard MB, Bhagat YA, Sun W, Magland JF, Wehrli FW (2012) Micro-MR imaging-based computational biomechanics demonstrates reduction in cortical and trabecular bone strength after renal transplantation. Radiology 262: 912–920. 10.1148/radiol.11111044 22357891 PMC3285225

[4] Nishiyama KK, Pauchard Y, Nikkel LE, Iyer S, Zhang C, McMahon DJ, et al (2015) Longitudinal HR-pQCT and image registration detects endocortical bone loss in kidney transplantation patients. J Bone Miner Res 30: 554–561. 10.1002/jbmr.2358 25213758

[5] Mikuls TR, Julian BA, Bartolucci A, Saag KG (2003) Bone mineral density changes within six months of renal transplantation. Transplantation 75: 49–54. 10.1097/01.TP.0000040600.14084.3B 12544870

[6] Naylor KL, Li AH, Lam NN, Hodsman AB, Jamal SA, Garg AX (2013) Fracture risk in kidney transplant recipients: a systematic review. Transplantation 95: 1461–1470. 10.1097/TP.0b013e31828eead8 23594857

[7] Nikkel LE, Hollenbeak CS, Fox EJ, Uemura T, Ghahramani N (2009) Risk of fractures after renal transplantation in the United States. Transplantation 87: 1846–1851. 10.1097/TP.0b013e3181a6bbda 19543063

[8] Jamal SA, West SL, Miller PD (2012) Fracture risk assessment in patients with chronic kidney disease. Osteoporosis International 23: 1191–1198. 10.1007/s00198-011-1781-0 21901475

[9] Uhlig K, Berns JS, Kestenbaum B, Kumar R, Leonard MB, Martin KJ, et al (2010) KDOQI US Commentary on the 2009 KDIGO Clinical Practice Guideline for the Diagnosis, Evaluation, and Treatment of CKD–Mineral and Bone Disorder (CKD-MBD). American journal of kidney diseases: the official journal of the National Kidney Foundation 55: 773–799. 20363541 10.1053/j.ajkd.2010.02.340

[10] Ketteler M, Block GA, Evenepoel P, Fukagawa M, Herzog CA, McCann L, et al (2017) Executive summary of the 2017 KDIGO Chronic Kidney Disease-Mineral and Bone Disorder (CKD-MBD) Guideline Update: what's changed and why it matters. Kidney Int 92: 26–36. 10.1016/j.kint.2017.04.006 28646995

[11] Akaberi S, Simonsen O, Lindergard B, Nyberg G (2008) Can DXA predict fractures in renal transplant patients? Am J Transplant 8: 2647–2651. 10.1111/j.1600-6143.2008.02423.x 18853956

[12] Yamaguchi T, Kanno E, Tsubota J, Shiomi T, Nakai M, Hattori S (1996) Retrospective study on the usefulness of radius and lumbar bone density in the separation of hemodialysis patients with fractures from those without fractures. Bone 19: 549–555. 8922656 10.1016/s8756-3282(96)00246-3

[13] Jamal SA, Gilbert J, Gordon C, Bauer DC (2006) Cortical PQCT Measures Are Associated With Fractures in Dialysis Patients. Journal of Bone and Mineral Research 21: 543–548. 10.1359/jbmr.060105 16598374

[14] Jamal SA, Hayden JA, Beyene J (2007) Low bone mineral density and fractures in long-term hemodialysis patients: a meta-analysis. Am J Kidney Dis 49: 674–681. 10.1053/j.ajkd.2007.02.264 17472850

[15] Schousboe JT, Shepherd JA, Bilezikian JP, Baim S (2013) Executive summary of the 2013 International Society for Clinical Densitometry Position Development Conference on bone densitometry. J Clin Densitom 16: 455–466. 10.1016/j.jocd.2013.08.004 24183638

[16] Shepherd JA, Schousboe JT, Broy SB, Engelke K, Leslie WD (2015) Executive Summary of the 2015 ISCD Position Development Conference on Advanced Measures From DXA and QCT: Fracture Prediction Beyond BMD. J Clin Densitom 18: 274–286. 10.1016/j.jocd.2015.06.013 26277847

[17] Muxí Á, Torregrosa J-V, Fuster D, Peris P, Vidal-Sicart S, Solà O, et al (2009) Arteriovenous Fistula Affects Bone Mineral Density Measurements in End-Stage Renal Failure Patients. Clinical Journal of the American Society of Nephrology 4: 1494–1499. 10.2215/CJN.01470209 19713298 PMC2736695

[18] Levey AS GT, Kusek JW, Beck GJ. (2000) A simplified equation to predict glomerular filtration rate from serum creatinine. J Am Soc Nephrol 11: 155A.

[19] Kannus P, Haapasalo H, Sankelo M, Sievanen H, Pasanen M, Heinonen A, et al (1995) Effect of starting age of physical activity on bone mass in the dominant arm of tennis and squash players. Ann Intern Med 123: 27–31. 7762910 10.7326/0003-4819-123-1-199507010-00003

[20] Ducher G, Bass SL, Saxon L, Daly RM (2011) Effects of repetitive loading on the growth-induced changes in bone mass and cortical bone geometry: a 12-month study in pre/peri- and postmenarcheal tennis players. J Bone Miner Res 26: 1321–1329. 10.1002/jbmr.323 21611970

[21] Min JY, Min KB, Paek D, Cho SI (2007) Side differences in the bone density of the distal radius and calcaneus in Koreans aged 4–86 years. J Clin Densitom 10: 184–188. 10.1016/j.jocd.2006.12.004 17485037

[22] Shin A, Choi JY, Chung HW, Park SK, Shin CS, Choi YH, et al (2004) Prevalence and risk factors of distal radius and calcaneus bone mineral density in Korean population. Osteoporos Int 15: 639–644. 10.1007/s00198-004-1587-4 15042282

[23] Barbour KE, Zmuda JM, Strotmeyer ES, Horwitz MJ, Boudreau R, Evans RW, et al (2010) Correlates of trabecular and cortical volumetric bone mineral density of the radius and tibia in older men: the Osteoporotic Fractures in Men Study. J Bone Miner Res 25: 1017–1028. 10.1002/jbmr.6 20200975 PMC3153367

[24] Sergi G, Perissinotto E, Zucchetto M, Enzi G, Manzato E, Giannini S, et al (2009) Upper limb bone mineral density and body composition measured by peripheral quantitative computed tomography in right-handed adults: the role of the dominance effect. J Endocrinol Invest 32: 298–302. 10.1007/BF03345715 19636194

[25] Muyshondt I, Lateur L, Van Roost G, Maes B (2003) Osteolysis induced by AV-fistula in idiopathic carpotarsal osteolysis. Nephrol Dial Transplant 18: 2185–2188. 10.1093/ndt/gfg331 13679502

[26] Tasbas BA, Yenidunya S, Hosaka Y, Morohoshi T (2003) Arteriovenous fistula and bone healing: experimental study in the rat. J Reconstr Microsurg 19: 395–400. 10.1055/s-2003-42636 14515233

[27] Karacan I, Aydin T, Ozaras N (2004) Bone loss in the contralateral asymptomatic hand in patients with complex regional pain syndrome type 1. J Bone Miner Metab 22: 44–47. 10.1007/s00774-003-0447-1 14691686

[28] Diekerhof CH, Reedt Dortland RW, Oner FC, Verbout AJ (2002) Severe erosion of lumbar vertebral body because of abdominal aortic false aneurysm: report of two cases. Spine (Phila Pa 1976) 27: E382–384. 12195081 10.1097/00007632-200208150-00026

[29] Aydogan M, Karatoprak O, Mirzanli C, Ozturk C, Tezer M, Hamzaoglu A (2008) Severe erosion of lumbar vertebral body because of a chronic ruptured abdominal aortic aneurysm. Spine J 8: 394–396. 10.1016/j.spinee.2006.12.001 18299106

[30] Mancini F, Ascoli-Marchetti A, Garro L, Caterini R (2014) Aseptic lysis L2-L3 as complication of abdominal aortic aneurysm repair. J Orthop Traumatol 15: 291–294. 10.1007/s10195-014-0308-9 25017025 PMC4244547

[31] Shen JI, Winkelmayer WC (2012) Use and safety of unfractionated heparin for anticoagulation during maintenance hemodialysis. Am J Kidney Dis 60: 473–486. 10.1053/j.ajkd.2012.03.017 22560830 PMC4088960

[32] Cruz DN, Wysolmerski JJ, Brickel HM, Gundberg CG, Simpson CA, Mitnick MA, et al (2001) Parameters of high bone-turnover predict bone loss in renal transplant patients: a longitudinal study. Transplantation 72: 83–88. 11468539 10.1097/00007890-200107150-00017

[33] Iyer SP, Nikkel LE, Nishiyama KK, Dworakowski E, Cremers S, Zhang C, et al (2014) Kidney transplantation with early corticosteroid withdrawal: paradoxical effects at the central and peripheral skeleton. J Am Soc Nephrol 25: 1331–1341. 10.1681/ASN.2013080851 24511131 PMC4033378

[34] Evenepoel P Recovery Versus Persistence of Disordered Mineral Metabolism in Kidney Transplant Recipients. Seminars in Nephrology 33: 191–203. 10.1016/j.semnephrol.2012.12.019 23465505

[35] Yenchek RH, Ix JH, Shlipak MG, Bauer DC, Rianon NJ, Kritchevsky SB, et al (2012) Bone mineral density and fracture risk in older individuals with CKD. Clin J Am Soc Nephrol 7: 1130–1136. 10.2215/CJN.12871211 22516286 PMC3386677

[36] Iimori S, Mori Y, Akita W, Kuyama T, Takada S, Asai T, et al (2012) Diagnostic usefulness of bone mineral density and biochemical markers of bone turnover in predicting fracture in CKD stage 5D patients—a single-center cohort study. Nephrol Dial Transplant 27: 345–351. 10.1093/ndt/gfr317 21652550

[37] Jamal S, Cheung AM, West S, Lok C (2012) Bone mineral density by DXA and HR pQCT can discriminate fracture status in men and women with stages 3 to 5 chronic kidney disease. Osteoporos Int 23: 2805–2813. 10.1007/s00198-012-1908-y 22297732

